# Postoperative cognitive deficit after cardiopulmonary bypass with preserved cerebral oxygenation: a prospective observational pilot study

**DOI:** 10.1186/1471-2253-11-7

**Published:** 2011-03-14

**Authors:** Axel Fudickar, Sönke Peters, Claudia Stapelfeldt, Götz Serocki, Jörn Leiendecker, Patrick Meybohm, Markus Steinfath, Berthold Bein

**Affiliations:** 1 Department of Anesthesiology and Intensive Care Medicine, University Hospital Schleswig-Holstein, Campus Kiel, Schwanenweg 21, D-24105 Kiel, Germany

**Keywords:** Monitoring, near infrared spectroscopy, cardiopulmonary bypass, cognitive symptoms

## Abstract

**Background:**

Neurologic deficits after cardiac surgery are common complications. Aim of this prospective observational pilot study was to investigate the incidence of postoperative cognitive deficit (POCD) after cardiac surgery, provided that relevant decrease of cerebral oxygen saturation (cSO2) is avoided during cardiopulmonary bypass.

**Methods:**

cSO2 was measured by near infrared spectroscopy in 35 patients during cardiopulmonary bypass. cSO2 was kept above 80% of baseline and above 55% during anesthesia including cardiopulmonary bypass. POCD was tested by trail making test, digit symbol substitution test, Ray's auditorial verbal learning test, digit span test and verbal fluency test the day before and 5 days after surgery. POCD was defined as a decline in test performance that exceeded - 20% from baseline in two tests or more. Correlation of POCD with lowest cSO2 and cSO2 - threshold were determined explorative.

**Results:**

POCD was observed in 43% of patients. Lowest cSO2 during cardiopulmonary bypass was significantly correlated with POCD (p = 0.015, r2 = 0.44, without Bonferroni correction). A threshold of 65% for cSO2 was able to predict POCD with a sensitivity of 86.7% and a specificity of 65.0% (p = 0.03, without Bonferroni correction).

**Conclusions:**

Despite a relevant decrease of cerebral oxygen saturation was avoided in our pilot study during cardiopulmonary bypass, incidence of POCD was comparable to that reported in patients without monitoring. A higher threshold for cSO2 may be needed to reduce the incidence of POCD.

## Background

Neurologic deficits after cardiac surgery are common complications with clinical manifestations ranging from stroke to subtle neurocognitive deficits [1].

Postoperative cognitive deficit (POCD) is a frequent complication of cardiac surgery with and without cardiopulmonary bypass (CPB) [2]. In a study by Newman and colleagues a significant cognitive decline, defined as a 20% reduction from baseline, occurred in 53% of patients at discharge, 36% at 6 weeks, 24% at 6 months, and 42% at 5 years [3]. Other authors report an incidence ranging from 8% to 40% [4].

Cognitive impairment is associated with lower general health after cardiac surgery with important implications for the care of patients undergoing cardiac surgery [5]. A retrospective examination of the influence of multimodal neuromonitoring on the incidence of serious brain injury associated with coronary artery bypass grafting (CABG) showed that in the absence of neuromonitoring the incidence of serious brain injury is twice as high as with neuromonitoring (6.1% vs 3.0%). The favourable results were attributed primarily to less non-embolic injuries in the neuromonitoring group [6]. Continuous cerebral oximetry is a relatively new technology for non-invasive brain monitoring [7]. Recently it has been shown by Murkin and colleagues that prolonged periods of low cerebral oxygen saturation (cSO2 <75% from baseline) during CPB are associated with a higher incidence of postoperative multiorgan dysfunction syndrome (MODS), longer intensive care periods and later discharge from hospital compared with patients treated immediately by readjustment of CPB parameters [8].

Cerebral oxygen desaturations are associated with early postoperative neuropsychological deficits in cardiac surgery and prolonged hospital stay [9]. Moreover, optimizing intraoperative cerebral oxygen delivery using noninvasive cerebral oximetry reduced the risk of stroke [10]. NIRS is used in 50% of all hospitals in the United States where pediatric heart surgery is performed and in 10% of all hospitals that provide adult heart surgery [11]. It was suggested that all cardiac surgical patients should have intraoperative cerebral oxygenation monitoring [12].

However, in a recent systematic review the predictive value of NIRS in identifying those who will suffer postoperatively from neurological deficits was questioned [13].

Thus it remains unclear, if maintaining acceptable cSO2 is able to prevent cognitive decline after CPB. We designed a CPB-protocol aimed at maintaining cSO2 above 55% and above - 20% from baseline during CPB. Main question was to investigate the incidence and severity of POCD in patients with cSO2 above 5% and above - 20% from baseline. The correlation of severity of POCD with lowest cSO2 during CPB was examined to analyse if cSO2 is associated with cognitive decline even if cSO2 is in the range regarded as acceptable.

## Methods

### Study design

Study design was prospective observational.

### Subject Enrollment

The study was approved by our local Human Investigations Committee (University Kiel, Schleswig-Holstein, Germany) and written informed consent was obtained from each patient. 35 patients undergoing cardiac surgery at the University Hospital Schleswig-Holstein, Campus Kiel, were enrolled in the study between March 2008 and November 2008. Patients with central nervous system disease, psychiatric disease, use of tranquilizers or antipsychotic drugs, drug abuse, visual, auditory or motor handicap, lacking knowledge of the German language or illiteracy were excluded from the study. Dementia, defined as a Mini-Mental-Test score below 24, was an exclusion criterion. Presence of unilateral or bilateral carotid stenosis was excluded by preoperative ultrasound Doppler examination of the carotid arteries.

### Neurocognitive Assessment

Neurological deficits were evaluated by routine clinical examination one day before surgery, at the intensive care unit after emergence from anesthesia and five days after surgery. Neuropsychological status was examined by a test battery consisting of trail making test (TMT) and digit symbol substitution test (DSST) for evaluation of attention, Ray's auditorial verbal learning test (AVLT) and digit span test (DST) for evaluation of memory and verbal fluency test (VFT) for evaluation of executive function including semantic and phonetic abilities one day before and **five **days after heart surgery [14]. A test score decrease of more than 20% from baseline was considered as clinically relevant. POCD was quantified as the number of tests with clinically relevant decrease of test score for each patient. A decline in performance from the preoperative test that exceeded - 20% from baseline in two tests or more was defined as neurocognitive deficit as described by Martens et al. [15].

### Intraoperative Procedure

All patients received midazolam (3.75 - 7.5 mg) per os preoperatively. From arrival in the anesthesia induction room, ECG and pulse oximetry were monitored continuously. Measurement of cSO2 was performed continuously from arrival at the anesthesia induction room to the end of CPB using a NIRO 300 Spectrometer (Hamamatsu Photonics K.K., Hamamatsu City, Japan). cSO2 was monitored unilaterally. Quality of data was verified by the automatic artefact recognition of the NIRO 300 Spectrometer, continuous evaluation of the measured parameters and continuous visual observation of the measured curve for artefacts. A decline of cSO2 below 80% of baseline or below 55% measured for at least one minute was regarded as relevant during anesthesia including cardiopulmonary bypass. A cannula was inserted in the left or right radial artery before induction of anesthesia to measure the blood pressure continuously. Anesthesia was induced with propofol (2 mg.kg-1) and sufentanil (0.5 μg.kg-1). Muscle relaxation was achieved with rocuronium (0.6 mg.kg-1). 90 s after injection of rocuronium, the trachea was intubated and the lungs were mechanically ventilated. Subsequently, anesthesia was maintained with sevoflurane (end tidal 1.5 - 1.7%) in an air/oxygen mixture (FiO2 = 0.5) and sufentanil (1 μg.kg-1.h-1). Vasoactive drugs were administered to maintain systolic arterial blood pressure between 90 to 120 mmHg after induction of anesthesia and mean arterial blood pressure was maintained above 60 mmHg. A central venous line was inserted into the right internal jugular vein to measure the central venous pressure.

Extracorporal circuit was primed with 1000 ml ringer 's lactate, 500 ml hydroxyethyl starch 6%, 200 ml mannitol 15% and 10000 IE heparin. Activated clotting time (ACT) was adjusted to values of more than 400 s by intravenous injection of heparin (300 IE.kg-1) before starting CPB. Initial pump flow was 2.5 l.min-1.m-2. Mean arterial pressure (MAP) was kept at 60 - 70 mmHg by adjusting pump flow up to 5.6 l.min-1.m-2. In addition to this, intravenous norepinephrine (0.02 - 0.2 μg.kg-1.min-1) or nitroglycerin (0.5 - 5 μg.kg-1.min-1) were used to control MAP when needed. pCO2 was managed using α-stat. Body temperature was cooled to moderate hypothermia (32° - 34°C). Catecholamines were routinely given, when mean arterial blood pressure was below 60 mmHg with maximal CPB flow (5.6 l.min-1.m-2).

Increasing CPB pump flow or mean arterial blood pressure due to low cSO2 was not needed during the study because cSO2 was always within the limits regarded as sufficient. Standard operating procedure provided for possible low cSO2 was checking head position, increasing pCO2 above 40 mmHg, increasing mean arterial blood pressure above 60 mmHg and increasing haematocrit above 25% by transfusion of erythrocytes. However, there were no study related interventions.

### Statistics

Statistical analyses were performed using commercially available software (GraphPad Prism 5.0, GraphPad Software, San Diego, USA) and free online software (Wessa, P. (2009) Free Statistics Software, Office for Research Development and Education,version 1.1.23-r4, URL http://www.wessa.net/). Correlation of lowest cSO2 during CPB with POCD was calculated using Kendall tau rank correlation coefficient test. Receiver-operator characteristic curves (ROC) were used to calculate sensitivity and specificity of cSO2-limits. P < 0.05 was regarded as statistically significant.

## Results

Demographic data are shown in table 1. Patients received surgery with use of CPB for aorto-coronary bypass and aortic or mitral valve repair (Table 2).

**Table 1:** Demographic data of patients (*Values are mean (SD)).

Age (yrs)	67.5 (10.9)*
Weight (kg)	77.1 (11.4)*
Height (cm)	172.8 (8.4)*

**Table 2:** Surgical procedures and number of patients.

Surgical procedure:	Number of patients
Aorto-coronary bypass	18
Aortic valve repair + Aorto-coronary bypass	6
Aortic valve repair	3
Mitral valve repair + Aorto-coronary bypass	3
Mitral valve repair	2

**Figure 1:**
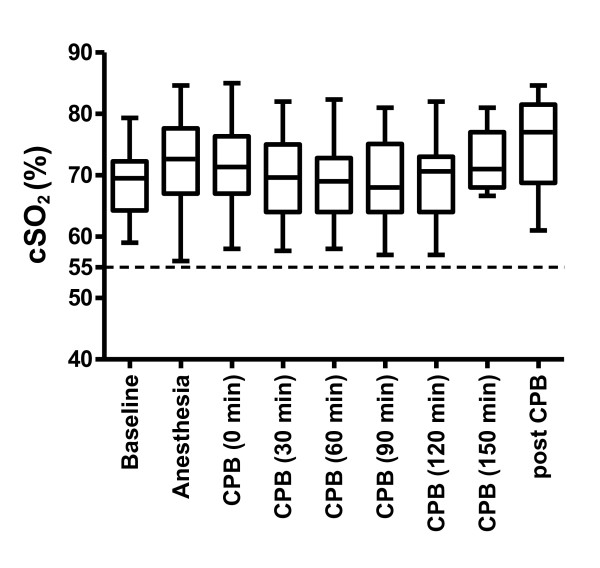
**Cerebral oxygen saturation (cSO_2_) at points of interest before induction of anaesthesia (Baseline), during anaesthesia before cardiopulmonary bypass (Anaesthesia), during cardiopulmonary bypass (CPB, 0 min - 150 min) and during anaesthesia after cardiopulmonary bypass (post CPB)**. The additional grid line shows the absolute lower limit of cSO_2 _(55%). Data is given as median, 25th/75th percentile and range.

No patient had relevant decrease of cSO2 from induction of anesthesia to the end of CPB (Figure 1). Also, no patient had focal neurologic deficit or apparent severe cognitive deficit on postoperative clinical exam. Cognitive testing revealed clinically relevant decline of performance in no test for 10 (28.5%), in one test for 10 (28.5%), in two tests for 6 (17%), in three tests for 7 (20%) and four tests in 2 patients (6%). Following the criteria for neurocognitive deficit, 15 patients suffered from POCD (43%).

**Figure 2:**
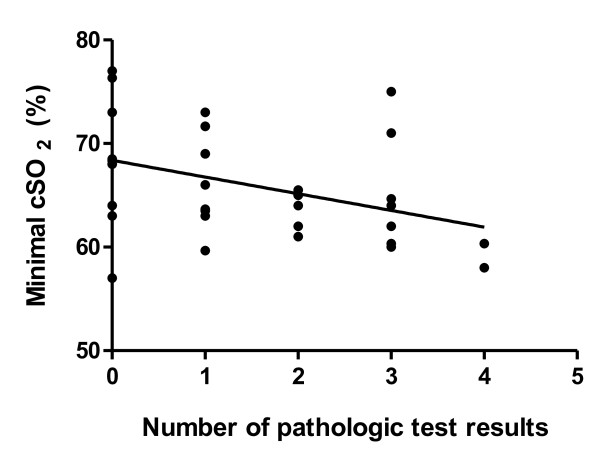
**Correlation of severity of postoperative neurocognitive deficit (POCD) defined as the number of tests with clinically relevant decline (decrease of postoperative test results below 80% of preoperative baseline) after cardiac surgery with minimal cSO_2 _during cardiopulmonary bypass by trend (p = 0.015, r^2 ^= 0.44, without Bonferroni correction)**. POCD was investigated by a set of five neuropsychological tests 1 day before and 4 days after heart surgery with cardiopulmonary bypass.

**Figure 3:**
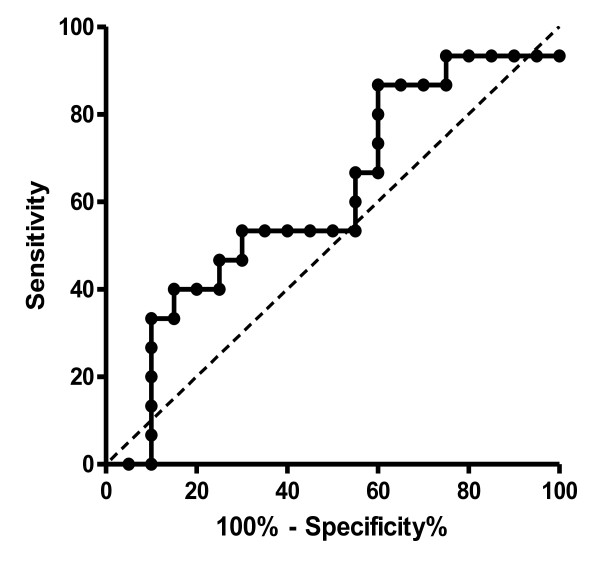
**Receiver-operator characteristic (ROC) curve of an absolute threshold of 65% for minimal cSO_2 _during cardiopulmonary bypass**. The threshold discriminates patients with and without postoperative neurocognitive deficit with a sensitivity of 86.7% and a specificity of 65.0% by trend (p = 0.03, AUC = 0.61, without Bonferroni correction). Dashed line is line of identity.

There was a significant correlation of the lowest cSO2 during CPB with POCD (r2 = 0.44, p = 0.015, figure 2). An absolute cSO2-threshold of 65% during CPB discriminated patients with and without POCD with a sensitivity of 86.7% and a specificity of 65.0% (p = 0.03, AUC 0.61, figure 3). cSO2-thresholds in relation to baseline values could not significantly discriminate between patients with and without POCD (p = 0.27).

## Discussion

### Basic Findings

Main findings of our prospective observational trial are as follows:

1. Although cerebral oxygen saturation was maintained above 80% of baseline and above 55%, incidence of postoperative cognitive deficit after cardiopulmonary bypass was 43%.

2. Lowest cSO2 during CPB correlated significantly with POCD (r2 = 0.44, p = 0.015).

3. An absolute threshold of 65% for cSO2 during CPB discriminated patients with and without POCD with a sensitivity of 86.7% and a specificity of 65.0% (p = 0.03).

### Near infrared spectroscopy (NIRS)

Monitoring NIRS cSO2 is regarded as a promising, but not yet widely accepted tool to reduce POCD. It has been evaluated in heart surgery as well as in non-cardiac surgery [16,17]. Normal values for cSO2 measured by near infrared spectroscopy have been calculated from 1000 patients with heart disease (67 ± 10%) and normal individuals (71 ± 6%). Baseline values depend from age, but not from weight, height, head circumference, sex, smoking or caffeine consumption. Baseline oxygenation values correlate positively with hemoglobin concentrations. Low baseline values are associated with poor neurological outcome and prolonged hospital stay [11].

Baseline values are varying considerably between individuals (47% to 83%) in other studies and normal values are not easily defined [13]. Clinical evaluation of brain tissue oxygenation by NIRS is usually limited to changes from baseline. A lower limit of 80% of baseline and an absolute lower limit of 50% are regarded as reasonable limits for cSO2 [11]. To improve safety an absolute lower limit of 55% was chosen in our investigation.

### Cognitive testing

Neurocognitive deficit was investigated according to the statement of consensus on assessment of neurobehavioral outcome after CPB [18]. Decline in test results of more than 20% in two or more tests was defined as POCD according to previous clinical investigations [15].

Cognitive testing was limited to baseline testing preoperatively and testing at day 5 postoperatively. However, cognitive function at discharge was a significant predictor of long-term function in a study designed to longitudinally investigate cognitive decline after cardiac surgery with CPB. Incidence of cognitive decline was found to be 53% at discharge, 36% at six weeks, 24% at six months, and 42% at five years in this study [3]. Consequently, testing at discharge was considered as sufficient to investigate clinically relevant postoperative cognitive deficit.

### Pocd

POCD was observed in 43% of all patients although decrease of cSO2 below 80% of baseline of cSO2 and below 55% was avoided. Hence, keeping cSO2 within these limits was not sufficient to reduce POCD in comparison to the median range of incidences reported in previous investigations [3,19]. Therefore, the generally accepted lower limit of cSO2 may be too low to avoid POCD. However, this conclusion remains questionable due to the small sample size. A larger sample size would answer this question.

ROC curves showed that an absolute threshold of 65% for cSO2 during CPB could discriminate patients with and without POCD with reasonable sensitivity and specificity in our investigation. Consequently, maintaining cSO2 above the threshold of 65% may reduce the incidence of POCD.

### Interventions

Standard operating procedures for possible low cSO2 in our study were designed according to Murkin et al. and results of a study on the effects of hemodilution during CPB on POCD [8,20]

However, study-specific interventions were not necessary in our patients, possibly because CPB parameters were optimized.

### Limitations of NIRS

During this study cSO2 was monitored unilaterally. Bilateral measurement of cSO2 would have been superior to unilateral measurement, because unilateral frontal perfusion deficits could have been missed by only one optode. Unfortunately, only one optode was available during the study. However, hypoperfusion during CPB is generally expected to affect both hemispheres and even with two electrodes, regional hypoperfusion or embolism far from the frontal optodes would have been missed by the measurement. Generally, therapeutic interventions prompted by decrease of NIRS derived cSO2 may not be sufficient to improve cerebral outcome in all patients due to the variety of potential mechanisms of brain injury contributing to POCD:

1. NIRS does not reveal all mechanisms of central nervous system injury and may miss embolism if the afflicted area is far from the optode site. Cerebral emboli are an important risk during cardiac surgery, even in patients who do not have clinically overt stroke [21,22]. Generally, NIRS is limited to the detection of general and forebrain ischemia and may miss injury that is not located in the measuring volume.

2. Global hypoperfusion injury may be aggravated by hyperthermia during rewarming unnoticed by NIRS monitoring.

3. Inflammation, activated during CPB, has been identified as a risk factor of POCD [23]. Specific proinflammatory genetic polymorphisms including CRP and IL-6 polymorphisms are associated with POCD, thus possibly explaining the variable susceptibility to POCD [24].

4. POCD after CPB may also be triggered by a preoperative decline of cognitive function [19]. In a subgroup of patients, preexisting cognitive deficit caused by cerebral vascular disease may be aggravated by embolism or hypoperfusion during cardiac surgery. After primary recovery, these patients may later show a progressive cognitive decline independently from the cardiac surgery [19,25].

It remains unclear to what extent the described mechanisms contribute to POCD and thus the role of NIRS in the effort to avoid POCD has still to be defined.

## Conclusions

Despite a relevant decrease of cerebral oxygen saturation (cSO2) was avoided in our study during cardiopulmonary bypass (CPB), incidence of POCD was comparable to that reported in patients without monitoring. The results of our pilot study support the hypothesis that the incidence of POCD is not improved by keeping cSO2 above - 20% from baseline and that a larger prospective randomized study on this issue is clearly warranted.

## Competing interests

The authors declare that they have no competing interests.

## Authors' contributions

AF and BB designed and coordinated the study and drafted the manuscript. SP carried out the intraoperative measurements and perioperative cognitive tests. GS and CS performed anesthesia and participated in intraoperative measurements. JL, PM and MS participated in the design and coordination. All authors read and approved the final manuscript.

## Pre-publication history

The pre-publication history for this paper can be accessed here:

http://www.biomedcentral.com/1471-2253/11/7/prepub
